# “Clinical trials are space travel”: Factors of psychological response to recurrence among oncologists enrolling patients in treatment optimization trials

**DOI:** 10.1002/cam4.6710

**Published:** 2023-11-10

**Authors:** Nicole Lynn Henderson, Courtney Andrews, Stacey A. Ingram, Lisa Zubkoff, Nadine Tung, Lynne I. Wagner, Lauren P. Wallner, Antonio Wolff, Gabrielle B. Rocque

**Affiliations:** ^1^ University of Alabama at Birmingham Heersink School of Medicine Birmingham Alabama USA; ^2^ GRECC, Birmingham Virginia Healthcare System Birmingham Alabama USA; ^3^ Beth Israel Deaconess Medical Center Boston Massachusetts USA; ^4^ Department of Social Sciences and Health Policy Wake Forest University Health Sciences Winston‐Salem North Carolina USA; ^5^ University of Michigan, Rogel Cancer Center Ann Arbor Michigan USA; ^6^ John Hopkins University Baltimore Maryland USA

**Keywords:** De‐escalation, personalized medicine, recurrence distress, treatment optimization

## Abstract

**Background:**

Cancer recurrence after treatment is a concern for patients and oncologists alike. The movement towards treatment optimization, with trials testing less than the current standard of care (SoC), complicates this experience. Our objective was to assess oncologists' psychological response to patient recurrence on optimization‐focused trials and identify factors that influence those experiences.

**Methods:**

Clinical oncologists participated in a semi‐structured interview regarding patient enrollment in treatment optimization trials. We identified factors that influence the degree of psychological response that the oncologist may feel after patient recurrence. Residual agreement analysis was used to identify whether differences in reported psychological response was associated with alternative emphases on identified factors.

**Results:**

Thirty‐six oncologists identified 20 factors spanning five major themes that affected their psychological response to patient recurrence. All oncologists expressed willingness to enroll patients in treatment optimization clinical trials; however, half indicated that they were more likely to experience a negative psychological response after a treatment optimization trial than after a traditional intensification trial, and a quarter reported that patient recurrence on an optimization trial would impact their recommendations for future trial enrollment. Oncologists who reported more negative psychological responses to patient recurrence after participation in an optimization trial were more likely to emphasize introspective factors, while those who reported no difference in response emphasized patient‐ and process‐focused factors.

**Conclusions:**

Although most oncologists recognize the importance of treatment optimization trials, a significant proportion indicated a greater potential for psychological distress following patient recurrence in such trials and offered insight into how trial design and the process of patient enrollment can be improved to minimize those negative psychological responses.

## 1 INTRODUCTION

In oncology, there is a movement to scale back treatment intensity with the goal of maintaining long‐term survival outcomes while improving quality of life.^1^ This contrasts with traditional intensification trials that add therapy to improve survival outcomes. This “optimization” (formerly referred to as “de‐escalation”, a term disliked by patients) strategy can be challenging for patients and oncologists, as there are potential physical and psychological consequences to “doing less” than the current standard of care (SoC). Our previous work exploring patient perceptions of treatment optimization trials found 43% of patients with breast cancer would be unwilling to participate in a treatment optimization trial that tested less intense treatment, with 85% citing fear of recurrence as a key barrier.^2^ This contrasted with 19% who felt that clinical trials themselves were a barrier.^2^

Although psychological consequences of treatment optimization trials have been previously explored in patient populations,^3^ less is known regarding the oncologist perspective, despite their leading role in treatment decision‐making. Mehlis and colleagues^4^ found high distress among health professionals who regularly made decisions to limit life‐prolonging treatment and suggested better integration of decisional support to ease the burden of these decisions. Here, we seek to better understand oncologist perspectives on optimization clinical trials, focusing on how various patient‐, provider‐, and trial‐based factors influence their potential psychological responses to patient recurrence. Furthermore, we identify several structural and procedural factors of optimization‐focused trial designs that could impact psychological response on the part of the oncologist.

## 2 METHODS

### 2.1 Participants

This qualitative sub‐study is part of a larger project evaluating oncologist perspectives on optimization‐focused clinical trials for breast cancer patients.^2^, ^5^ Purposive sampling techniques were utilized to identify a balanced convenience sample of oncologists according to setting, gender, ethnicity, age, years of experience, and geographic location. In‐depth sample methodology and oncologist characteristics are described elsewhere.^6^ Briefly, physicians from different US practices were identified through working relationships with the oncologists, engagement with the ECOG‐ACRIN Breast Committee, or referral from previous participants. All academic physicians were breast cancer specialists, while community oncologists treated a spectrum of cancer types. Prior to the interviews, participants completed a questionnaire including basic demographic information and practice characteristics.

### 2.2 Interviews

Interviews were conducted by a breast medical oncologist (GR) via Zoom or telephone using a semi‐structured interview guide developed utilizing an a priori model‐based Norton and colleague's De‐implementation Framework^7^ and aligned with patient interview guides from a prior study focused on patient perspectives of optimization trials.^2^ The full interview guide has previously been published along with overarching barriers and facilitators that oncologists perceived to enrolling breast cancer patients in optimization trials.^6^ This analysis delves deeper into a subsection of the interview where oncologists were asked how they would feel if a patient recurred after participating in an optimization trial. The interviewer probed specifically to determine (1) whether negative psychological responses on the part of the oncologist occurred after patient recurrence; (2) whether negative psychological responses would be intensified by patient enrollment in an optimization trial rather than a traditional intensification trial; and (3) whether experience of patient recurrence would affect their future decision‐making regarding patient enrollment in treatment optimization trials. Then, additional free‐listing‐style questions were utilized to identify how physicians managed their psychological responses.^8^, ^9^ The goal was to better understand the shared experience of responding to patient recurrence by identifying the broad range of patient‐ and case‐related factors that could potentially impact oncologists' emotional/psychological response.^10^ All interviews were 30–60 min in length.

### 2.3 Analysis

#### 2.3.1 Freelisting and thematic analysis

Interviews were recorded, transcribed, and uploaded to QSR International's NVivo 11.4.3 for analysis. Two independent coders (NLH and CJA) performed inductive content analysis, identifying factors impacting the degree of psychological response and collating them into an ordered freelist for each physician. An iterative process of synonym identification and factor simplification then occurred through cross‐coder discussion with input from the interviewer (GBR). The final list of factors was reviewed and agreed upon for each physician by both coders and the interviewer. These freelists were temporally ordered, with factors mentioned earlier appearing higher on the list than factors mentioned later. This technique of qualitative data analysis allows for a determination of item (factor) salience, Smith's *S*, which is calculated using a combination of how often specific items were discussed by informants and when in the context of the interview they were discussed.^11^ Smith's *S* index is defined as *S* = ((L−Rx + 1)/L)/N, where L is the length of each list, Rx is the rank of item X in the list, and N is the number of lists in the sample. The index generates values between 0 and 1, with values closer to 1 indicating greater salience according to how often and early a specific factor was mentioned.

The list of factors for each interview participant was analyzed using FLARES v. 1.0 (Free List Analysis under R Environment using Shiny).^12^ FLARES assesses data saturation by mapping the number of newly identified themes as the number of respondents increases and comparing these figures to a logarithmic trend line. When good fit exists between the data and the trend line, data saturation is reached.^13^ Additionally, FLARES calculates the frequency of mention of each factor by participants and Smith's *S*. Factors discussed by at least 10 percent of the sample or that had a Smith's *S* of 0.10 or higher were retained for further analysis as these were the considerations that were most important to our participants.^11^, ^12^

The analytic team organized factors into major thematic groupings. These groupings were determined according to inductive thematic analysis and completed a priori to and separate from the following residual agreement analysis. Frequencies were also calculated for thematic groupings by calculating whether each individual mentioned any of the factors associated with a particular theme. Smith's *S* was not calculated for themes as FLARES does not analyze at this level of data abstraction.

#### 2.3.2 Residual Agreement Analysis

In order to identify how factors were patterned and distributed within our sample, a novel application of Dressler and colleagues' residual agreement analysis (RAA) was performed on the freelists.^14^ Traditionally, RAA has been performed following the final phase of cultural domain analysis to identify alternative perspectives within a shared cultural model.^8^, ^9^ Cultural models are simplified mental frameworks that people utilize to engage with and participate in the social world around them; they are shared understandings about “ways of doing things” that allow individuals in a group to live concordantly and harmoniously.^15^, ^16^, ^17^, ^18^, ^19^, ^20^ Traditional cultural model theory would posit that due to the numerous shared experiences involved in medical training and practicing medicine, oncologists would share a mental schema for rationalizing and responding to patient recurrence after clinical trial participation.

More recently, there has been a greater appreciation for how disagreement and alternative experiences are patterned within a defined population.^21^, ^22^ In other words, there are shared experiences (e.g., patient recurrence after treatment) that involve shared considerations (e.g., factors that impact psychological/emotional response) that may be idiosyncratically employed by individuals. In this case, we sought to determine whether individuals who experienced greater emotional or psychological responses after patient recurrence on an optimization trial discussed decisional factors in the same manner as those who did not perceive a difference between optimization and traditional trials.

First, we coded the responses to probe #2 (whether an individual's negative psychological response would be intensified by patient enrollment in an optimization trial rather than a traditional intensification trial) into a dichotomy. Subgroup 1, the “equal” group, stated that there were no differences in their personal psychological response to patient recurrence in cases of traditional or optimization trial enrollment. Subgroup 2, or the “more” group, then included those who reported that they would experience a greater negative psychological response to patient recurrence on a treatment optimization trial than on a traditional trial. Frequency (number of mentions/sample size) for each factor was calculated for the total sample and for each subgroup. The total sample frequency was subtracted from each of the subgroup frequencies, producing item residuals representing whether that particular subgroup was more or less likely to mention a factor than the overall sample. The residuals were plotted to identify where deviations occurred and to guide exploration into how oncologists differentially grapple with the potential for patient recurrence.

## 3 RESULTS

In total, 46 individuals were approached, and 39 ultimately participated in the interview. Refusals were not patterned according to any documented demographic factors and were primarily due to scheduling difficulties. Due to time constraints within interviews themselves, three individuals were not asked questions related to their emotional/psychological responses to patient recurrence and were excluded from this sub‐study analysis. Thus, a total of 36 oncologists were included in this analysis, and Table 1 presents participant demographics for the sample. The overall sample was balanced across gender (51% female), institutional affiliation (51% academic), oncologist age (*m* = 51.6; SD = 11.3), and years of experience (*m* = 18.4; SD = 12.2). The sample was evenly split regarding career stage, with 33% having less than 10 years of experience, 33% having between 10 and 20 years of experience, and 33% having over 20 years of experience.

**TABLE 1:** Descriptive statistics of the sample.

	Total sample	Subgroup 1: “Equal Distress”	Subgroup 2: “More Distress”
Sample size	36	17	19
Gender (%F)	51.4	52.9	47.4
Ethnicity: %white	75.7	70.6	78.9
%Black	8.1	11.8	5.3
%Hispanic	5.4	0	10.5
%Asian	10.8	17.6	5.3
Age (*m*, SD)	51.6 (11.3)	54.7 (11.5)	49.4 (10.9)
Years of practice (*m*, SD)	18.4 (12.2)	20.5 (12.4)	17.1 (12.1)
Institution type (%Aca)	51.4	41.2	57.9
%Would feel recurrence stress	69.4	64.7	100
%Recurrence would affect future decisions	25.0	13.3	35.3
Factor of distress list length (*m*)	7.1	6.1	7.9

### 3.1 Factors contributing to physician psychological response

Twenty factors that had the potential to impact psychological response were mentioned by at least 10% of the sample (>3 oncologists) and fell under five key themes: regulatory concerns and trial specific factors, oncologist emotions, practice of medicine, communication, and tailoring to the patient. Table 2 summarizes these key themes and individual factors, providing frequencies, Smith's *S*, and exemplary quotes where appropriate. There was a very good fit (*R* ^2^ = 0.989) between the data collected and the logarithmic trend line, indicating that the sample was large enough to achieve response saturation.^13^

**TABLE 2:** Major themes, factors of regret, and exemplary quotes.

Theme	Freq		Factor	Freq	Smith's *S*	Exemplary quotes
Regulatory concerns and trial Specific factors	0.97	MD 1: “There were lots of smart people who designed this trial and looked at the biology and felt like this was an appropriate decision for you. And it was approved by IRB, all these other things, because it's not just you as a doctor doing something random. We presented this trial ad nauseum into groups of experts and the design was careful. I think that would allow me to not be plagued at night by decisional regret.”	Importance of dropped medication	0.50	0.3684	MD 7: “But I guess I would feel less [regret] if it was a more minor switch, like dropping carbo from the HER2 type of regimen, which I perceive as minor, because I know some people will have metastatic recurrence even if they got all the treatment.”
			Trial design	0.44	0.2983	MD 24: “And, hopefully, the study has been thoughtfully designed where there's interim analysis and we can look at our data as we go along. And, I think that's very important in de‐escalation designs.”
			Trial rationale	0.39	0.2524	MD 14: “I think you have to make an objective decision whether the trial makes sense and whether it's what you believe in and then you have to separate the objective decision from emotional decisions.”
			Rigor of the process	0.19	0.0856	MD 8: “That's where the DSMB and the PI can be so helpful to say, “Hi, I know you have been burned. I've asked the DSMB to see if we have any early recurrence. I'm going to look at the data and if there's a problem, I will let you know” and if it was you that told me that, I believe you and trust you to do it.”
Practice of medicine	0.86	MD 14: “Well, I mean, again, when you practice long enough stuff happens to your patients. And I think if you are a good clinician, you care a lot about your patients. You always wonder if how you intersected with their destiny cost them their life. And certainly, clinical trials are space travel. It's dangerous. Does not mean you do not do it. And it does not mean it's not for the bigger good, but I think if you are asking if an astronaut dies along the way and you are in charge of them, it's still painful even if you are expecting it, so sure.”	Recur anyway	0.75	0.3525	MD 1: “Patients recur no matter what we do. And I think in order to continue to march forward and do your job, you sort of take those things a little bit in stride.”
			Nature of clinical trials	0.47	0.2565	MD 4: “So, it's something I have to live with and it's not going to stop me from writing clinical trials or enrolling patients in clinical trials because shit happens.”
			Social importance of trials	0.22	0.0786	MD 7: “Making bigger changes in our regimens is going to get us to where we need to go as a field faster, and it's going to ultimately have a larger impact on the community.”
			Compartmentalization	0.11	0.0508	MD 1: “I think the way that I sort of deal with this and allow myself to sleep at night and go on and be a human being is to try to compartmentalize it and say to myself “I did the best I could and these things happen.”
Oncologist emotions	0.83	MD 17: “Now imagine if you are the one that omitted that. So you will, of course feel, unless you are not human, unless… So I will for any of my patients that relapse, I go and look why. What happened? Did we wait too long to start treatment? Were we treating metastatic disease when we started and think we are doing adjuvant? Should we have done imaging to make sure this was not… So yes, I personally will feel now is that, wrong or right? But that's just a personal feeling about my patients.”	Human nature	0.50	0.3843	MD 12: “I think just by human nature, you are going to feel regrets.”
			Did I do enough?	0.39	0.2569	MD 24: “Because, at least you did not sacrifice the standard. You threw the kitchen sink, and it did not work out. It sucks, but it sounds like you cut a corner and are paying the price.”
			Oncologist mindset and biases	0.39	0.2065	MD 31: “At that point they asked me what I would do and I said, “You're young and I do not think I would do it.” So they did not… Like I said, she had this relapse in the bones… But I did not push that person to do it because they had that path CR and I had that bias and they wanted another child because they are coming of age soon.”
			Second thoughts	0.28	0.1936	MD 12: “I think it gives you second thoughts about how actively you are going to participate in a trial when people relapse.”
			Role of anecdote	0.28	0.1135	MD 13: “Yeah. I had a teacher once who always reminded me that the anecdote was the lowest form of science, but it's almost humanly impossible not to look at a string of successes and failures in a clinical trial and begin to wonder out loud whether the results are what you are seeing.”
			Negative trial	0.17	0.1146	MD 29: “Yeah, the answer to that question would be what the ultimate outcome of the trial was. So, if I knew that in that trial, that the patients did really well, even without therapy, then I'd be less likely to beat myself up.”
Communication	0.69	MD 28: “I think if I've done my job, if I've brought equipoise to the table at the time of the conversation and I've really done my best to make the patient have what I believe to be an informed decision, as informed as it can be, and they are part of the plan and they understand that there's inherent risk, and God forbid the recur, I would not feel that I have done them wrong.”	Right Decision at Time	0.50	0.2519	MD 26: “We did what we thought we should, with the best available information we had at the time.”
			Informed consent	0.33	0.1968	MD 30: “When they signed up for the trial, that was described in the consent form. It was a risk. It was something that was unlikely, but not impossible.”
			Shared decision making	0.33	0.1789	MD 7: “I think it makes me feel a little less bad in that it was a shared decision that we undertook the risk of those unknowns.”
Tailoring to the patient	0.56	MD 31: “And at that point had a path CR, and was still on the fence about having a third child. So that person decided to do Tamoxifen alone after they finished up Herceptin, they gave themselves a year or two to think whether you are going to have a child or not. I thought that was reasonable because they were still at the age where they had to make decisions.”	Disease biology	0.31	0.2024	MD 5: “So for me personally, my recurrences, the early‐stage patients that recur is the worst part of my job. Those are people that you treated with curative intent, and you feel like your treatment wasn't good enough.”
			Better life quality	0.28	0.1055	MD 3: “The other way to think about it is, this person is going to have recurrence and you saved her a year of her life that was so much better than if she'd been getting chemotherapy.”
			Patient characteristics (age, personality)	0.22	0.0801	MD 6: “It all comes back to the personality of the patient. If I feel like the patient is someone who is going to herself have regrets, then I'm going to have regret, yes. So, but that would be the kind of person that I probably would not put on the trial in the first place.”

#### 3.1.1 Regulatory and trial specific concerns

Regulatory and Trial Specific Concerns referred to the trial design itself as well as to the researchers and institutions involved in its conceptualization and operation. Specific factors included the *importance of the dropped medication*, *the trial design*, *the rigor of the process*, *and/or the trial rationale*. Half the sample stated that the degree of change from SoC impacted their psychological response, with many being uncomfortable with more significant changes, such as dropping chemotherapy completely in the triple‐negative population. Oncologists also commonly (44%) preferred trial designs that allowed the “ability to rescue” patients with additional treatment who had not achieved a desirable biomarker result (e.g., complete pathologic response after neoadjuvant treatment). Still, there was some fear of becoming “too reliant” on biomarkers, as the tests were imperfect predictors of response. Seven oncologists (19%) discussed the importance of the *rigor of the process*, including being able to rely on the oversight of Institutional Review Boards (IRBs) and Data Safety and Monitoring Boards (DSMBs). Finally, *trial rationale* referred to the importance of oncologists understanding and belief in the trial itself (39%). Oncologists recognized that despite various mechanisms in place to ensure the highest level of safety of clinical trials, responsibility still fell on themselves to be familiar with the background literature and study protocols prior to enrolling patients.

#### 3.1.2 Practice of medicine

The next set of factors (practice of medicine) referred to the social importance of clinical trials as well as the role of rationality and equipoisality while treating patients that applies to trials as a whole rather than specific trials. This was the second most popular category, with 86% of participants mentioning at least one of these factors: *recur anyway*, *the nature of clinical trials*, *the social importance of clinical trials*, and *oncologist compartmentalization*. Three‐quarters of participants mentioned that the patient could have “recurred anyway” regardless of participation in a trial or receipt of SoC. This was because, “statistically speaking,” it is simply impossible to expect a positive outcome for every patient. This inherent “danger” and “uncertainty” involved in the *nature of clinical trials* was mentioned by nearly half the sample (48%). Still, there was recognition that clinical trials were “important” and “needed to be done” in order to provide benefits and the “best care possible” to the “greatest amount of people” (22%). As one oncologist said, “clinical trials are space travel. It's dangerous. Doesn't mean you don't do it. And it doesn't mean it's not for the bigger good, but I think if you're asking if an astronaut dies along the way and you're in charge of them, it's still painful even if you're expecting it.” Here, there is a direct contrast between the potential “sacrifice” of the individual with the “greater good.” To handle this dichotomy, oncologists practice *compartmentalization* (11%), separating the “objective decision from emotional decisions” to the best of their ability. This compartmentalization occurs constantly, but there was particular emphasis on maintaining “emotional space” from the patient when making treatment decisions.

#### 3.1.3 Oncologist emotions

At the same time, oncologists commented that this separation is often “easier said than done.” Oncologist emotions arose as the third most commonly discussed theme (83%). This theme emphasized the impossibility of fully separating the “humanity” of the oncologist from their work and provides direct contrast to the traditional stoic and “unemotional” view of the practice of medicine. Factors included *Human Nature*, *Did I Do Enough?*, *Oncologist Mindset and Biases*, *Second Thoughts*, *the Role of Anecdote*, and/or *the Potential for a Negative Trial*. Half argued that it was “natural” to experience psychological distress after patient recurrence, as caring for others is part of “who we are as human beings.” Still, the extent to which the individual felt regret was also impacted by personal feelings regarding whether they treated their patients to the best of their ability. Many (39%) oncologists discussed taking comfort in “doing everything possible” in SoC and intensification clinical trial contexts, as, at least in those cases, “you didn't sacrifice the standard” or “cut any corners.” More than a third (39%) raised the concepts of “mindsets” and “biases,” with several discussing their “risk tolerance” and how their personal inclination towards “playing it safe” or “being aggressive” had the potential to impact their treatment recommendations and how they felt about them long term. Oncologists also felt that these tendencies could be associated with age and years of experience, as many mentioned that they “used to” have negative psychological responses but “got over that a long time ago.” Another oncologist said, “I do think that people earlier in their training, until they've seen toxicities, are oftentimes a little more prone to treat than not. I have trained a bunch of people and watched a lot of people mature in their careers, they're always less aggressive over time.”

Three factors—having *second thoughts* (28%), *the role of the anecdote* (28%), and *the potential of a negative trial* (17%)—addressed oncologists' struggles with confronting familiarity bias. Several agreed that personally knowing someone who recurred after a trial would lead them to question their continued involvement in the trial. Although there was recognition that anecdotes are the “lowest form of science,” they also noted that personal stories have “power,” and ignoring them has the potential to dehumanize the practice of medicine. Oncologists were particularly concerned with recurrences if there were “several in a row,” as this could indicate that the trial itself was negative and all enrolled patients are potentially in danger of receiving sub‐par treatment.

#### 3.1.4 Communication

The final two themes shifted the focus away from the oncologist and their practice of medicine to the importance of communication with patients (69%) and the use of patient characteristics and preferences to tailor treatment (56%). The communication theme included three factors (*right decision at the time*, *informed consent*, and *shared decision‐making*) that emphasized patient agency and their understanding of their disease and treatment options. Many oncologists were able to “fall back on” the recognition that they and their patients made the “*right decision at the time*” (50% PoM), given their understanding of the situation at hand. For example, one oncologist said,

“I often say that there has really been no one in my career where I've omitted chemotherapy and I've regretted doing it. Does that mean that there's been nobody that's had a recurrence in the first five years, which is when I think you're going to have a chemotherapy effect with at least ER‐positive disease? Is there no one who's recurred? No, but if I were put in the same situation again, I'd make the same decision with the patient."

In other words, having a clear understanding of the patient's situation and a strong rationale for selecting a certain regimen were incredibly important for moderating potential future negative psychological responses to patient recurrence. Similarly, obtaining *informed consent* from patients (33%) and engaging in *shared decision‐making* (33%) aided in distributing the onus of responsibility among all participating parties. As discussed above, oncologists are acutely aware of the potential dangers of clinical trials, and therefore it is both “comforting” and “affirming” when the patient is an informed and active participant in treatment discussions.

#### 3.1.5 Tailoring to the patient

Oncologists also saw the potential for utilizing treatment optimization trials as a mechanism for tailoring patient care and generally felt more comfortable if decisions were made based on the individual's characteristics. Thus, this final theme, tailoring to the patient (56%), reflects the broader movement within medicine to provide patients with individualized treatment plans that are specific to them and their needs. This theme included three factors: *disease biology*, *better interim life quality*, and *patient characteristics*. *Disease biology* (31%) was a major consideration, as treatment determination depends heavily on size, stage, and the sub‐type of the individual's cancer. Some oncologists noted that they were hesitant to include middle‐stage (II or III) cancer patients in treatment optimization trials, as providing less treatment could lessen the chance for a complete cure. In contrast, larger, more aggressive cancers that were less likely to be cured were more appropriate for treatment optimization trials, as decreased treatment intensity could provide a marked increase in the patient's quality of life. In these cases, oncologists recognized that regardless of recurrence potential, an optimization approach could provide the patient with additional time where they were not as physically or emotionally taxed by overtreatment (28%). This improved quality of life may also help the patient tolerate and respond to recurrence‐related treatment and lead to better outcomes later on. Other patient characteristics, such as age and personality, were also discussed by 22% of oncologists. For example, younger patients and those that had “more to lose” were more likely to cause distress to the oncologist in the case of recurrence. Thus, these patient characteristics provided additional “rationale” for why specific decisions were made and would increase confidence in the decision and reduce the potential for negative psychological responses down the road.

### 3.2 Residual agreement analysis results

To investigate how discussion of these factors was distributed among the sample, the sample was dichotomized according to how patient recurrence would impact them. Subgroup determination was performed independently by two coders (NLH and CJA), and agreement was 100%. Seventeen oncologists reported no psychological difference (“equal”) between treatment optimization trials and intensification trials, and 19 reported they would experience a greater negative psychological outcome (“more”) after recurrence if a patient was on a treatment optimization trial. For example, one oncologist that was sorted into the “equal” subgroup stated, “It [patient recurrence] is always a worry. I think that you have to just put it in that context, and when it happens, acknowledge that that was the situation.” In other words, this individual recognizes that they will experience an emotional response to patient recurrence, but that response is not necessarily different based on the type of clinical trial that the patient had participated in. In contrast, other oncologists felt the brunt of the responsibility for reducing the amount of treatment that a patient received: “If they go through all this, we try to salvage them, and they have a bad outcome, yeah, I would regret it, yes. I would feel that I hadn't done something in that patient's best interest.”

Individuals who reported “more” potential distress were more often white, younger, had fewer years in practice, and worked in academic settings. There were statistically significant differences in the reported experiences of negative psychological outcomes, with 100% of the “more” subgroup reporting that they would experience a negative response in any case of patient recurrence compared to only 64.7% in the “equal” subgroup [*χ* ^2^ (1, *N* = 36) = 17.7, *p* < 0.001]. Furthermore, although numerically more individuals in the “more” subgroup reported that patient recurrence after a trial enrollment would affect their decision to continue enrolling patients in optimization‐focused trials, this difference was not statistically significant [35.3% vs. 13.3%; *χ* ^2^ (1, *N* = 36) = 2.1, *p* = 0.152].

**FIGURE 1:**
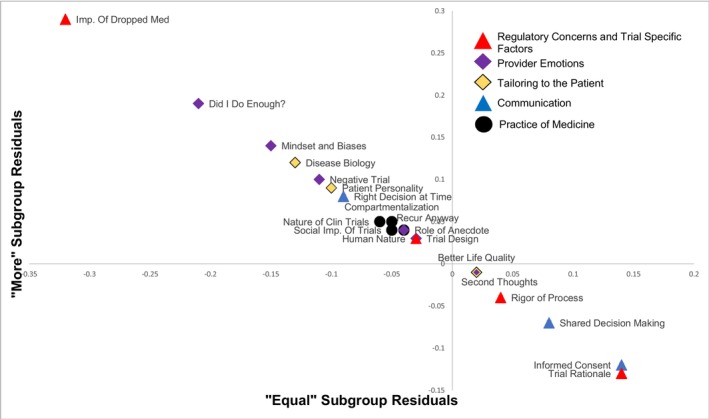
Residual agreement analysis of factor employment by reported experience of recurrence stress.

Figure 1 presents the results of the residual agreement analysis. The “equal” subgroup, located on the x‐axis, was more likely to discuss the *patient communication* and *regulatory concerns and trial‐specific factors* (located to right of the origin) and less likely to discuss *oncologist emotions* and *tailoring to the patients* (left of the origin). The subgroup that reported a greater negative response to optimization trial recurrences was the direct opposite, in that they emphasized their individual role and responsibility in the treatment process. Both groups had similar amounts of discussion related to the *practice of medicine*, as denoted by their central location in Figure 1.

## 4 DISCUSSION

We have demonstrated that the psychological impact of enrolling patients in treatment optimization clinical trials on oncologists is real and has the potential to influence future enrollment in optimization‐focused clinical trials. Although all oncologists in the study expressed interest in enrolling patients, a significant proportion noted hesitancy with regards to continued enrollment if one of their patients experienced a recurrence on a treatment optimization trial. In fact, this could be contributing to why several optimization trials struggled to recruit and retain patients. For example, the PIVOT trial, which randomized patients with prostate cancer to radical prostatectomy versus observation required 8 years (1994–2004) and 52 enrollment sites in order to meet enrollment goals.^23^ Only 15% (731/5023) of eligible and approached patients agreed to participate. Limited data on patient perspectives and reluctance exist^2^ and, to our knowledge, this is the first study that has approached enrollment hesitation from the oncologist point of view.

The factors alleviating negative psychological outcomes spanned five broad themes that represented facets of the trial itself: the practice of medicine, oncologist emotions, communication with patients, and the tailoring of treatment to individual patients. These factors act as psychological tools in the oncologist's arsenal for managing their emotional responses when first deciding which path is most appropriate for the patient and then later reflecting on whether the correct decision was made. Every factor may not be applicable in every patient's situation, but oncologists have developed a series of rationales that may be employed to better cognitively manage any potential or actual adverse events that result from treatment decisions.

It is important to note, however, that there were some differences regarding the emphases of factors. The subgroup of oncologists who emphasized their personal role in decision‐making process were more likely to experience psychological distress after patient recurrence than oncologists who emphasized regulatory mechanisms of the trial and proper consenting of patients. Interestingly, a larger proportion of academic oncologists were in the former group, reporting a greater likelihood of distress and emphasizing their personal role in the clinical trial development process. It is possible that because these academic oncologists *are* a part of those very regulatory mechanisms at their institutions, the importance of a checks and balances system has placed greater responsibility on their shoulders. In contrast, community‐based oncologists may enroll patients in trials but are less likely to have a continued role in ensuring trial safety.

Not all optimization‐focused strategies will result in outcomes equivalent to SoC, and oncologists must be prepared for the potential of a negative trial. Our study provides insight into how optimization‐focused trials could be designed in order to moderate negative psychological responses to patient recurrence on the part of the oncologist. First, larger changes to protocols were more likely to cause distress, meaning that optimization trials that involve smaller alterations to the SoC are likely to be more appealing. This is especially the case in clinical contexts where oncologists are already “doing less,” as cuts to standard protocols can feel more substantial. Second, most oncologists in the study appreciated the opportunity to “rescue” patients at intermediate stages of the trial. This built‐in “re‐evaluation” stage allows oncologists to re‐assess the patient's treatment process and affirm that the “right” decision was made or change course if the patient is not having a good response.

## 5 LIMITATIONS

The limitations of this study are similar to those of other exploratory and qualitative research projects.^24^ Coders worked in collaboration (NLH; CA) with the interviewing oncologist (GR) to ensure consistency and agreement regarding all codes. Furthermore, the small sample size could suggest that the results are not representative of the oncologist population across the United States. However, participant identification was careful and thorough as far as balancing gender, age, experience, location, and affiliation type, and there was no indication that oncologist responses differed significantly according to these characteristics. Additionally, as data saturation was achieved after only 4 participants, it is clear that oncologist responses were highly similar to each other, and additional respondents likely would not have added new perspectives to consider.

## 6 CONCLUSIONS

In this paper, we delved into oncologist's experience of potential negative psychological outcomes after patient recurrence in the optimization‐focused clinical trial setting. A broad range of factors impact the experience of negative feelings following patient recurrence. However, much of that distress can be addressed through ensuring careful design of the clinical trials themselves and greater involvement of the patient in the decision‐making process.

## AUTHOR CONTRIBUTIONS

**Nicole Lynn Henderson:** Formal analysis (lead); methodology (lead); writing – original draft (lead); writing – review and editing (lead). **Courtney Andrews:** Data curation (supporting); formal analysis (supporting); writing – original draft (supporting); writing – review and editing (supporting). **Stacey A. Ingram:** Data curation (supporting); funding acquisition (supporting); project administration (lead); resources (lead); supervision (supporting); writing – review and editing (supporting). **Lisa Zubkoff:** Conceptualization (supporting); methodology (supporting); writing – review and editing (supporting). **Nadine Tung:** Conceptualization (supporting); methodology (supporting); writing – review and editing (supporting). **Lynne I. Wagner:** Conceptualization (supporting); writing – review and editing (supporting). **Lauren P. Wallner:** Conceptualization (supporting); methodology (supporting); writing – review and editing (supporting). **Antonio Wolff:** Conceptualization (supporting); funding acquisition (supporting); methodology (supporting); supervision (supporting); writing – review and editing (supporting). **Gabrielle B. Rocque:** Conceptualization (lead); data curation (lead); formal analysis (supporting); funding acquisition (lead); investigation (lead); methodology (lead); project administration (supporting); resources (lead); supervision (lead); writing – original draft (supporting); writing – review and editing (supporting).

## FUNDING INFORMATION

Grant No. SAC170001 from the Susan G. Komen Breast Cancer Foundation Inc. d/b/a Susan G Komen and Grant No. T32 CA47888, Cancer Prevention and Control Training Program grant, from the National Cancer Institute, National Institutes of Health.

## CONFLICT OF INTEREST STATEMENT

Henderson (None); Andrews (None); Ingram (None); Zubkoff (None); Tung (Consulting: Astra Zeneca); Wagner (None); Wallner (Consulting: Gilead); Wolff (None); Rocque (Research: Pfizer, Genetech; Consulting: Pfizer, Flatiron, Gilead; Travel: Gilead).

## ETHICS STATEMENT

This study was approved by the University of Alabama at Birmingham Institutional Review Board.

## PATIENT CONSENT

Informed consent was obtained electronically for all participants.

## Data Availability

The data that support the findings of this study are available from the corresponding author upon reasonable request.

## References

[1] Wolff AC , Tung NM , Carey LA . Implications of neoadjuvant therapy in human epidermal growth factor receptor 2–positive breast cancer. J Clin Oncol. 2019;37:2189‐2192. 31157582 10.1200/JCO.19.01159

[2] Rocque GB , Williams CP , Andrews C , et al. Patient perspectives on chemotherapy de‐escalation in breast cancer. Cancer Med. 2021;10:3288‐3298. 33932097 10.1002/cam4.3891 PMC8124110

[3] Oh H‐M , Son C‐G . The risk of psychological stress on cancer recurrence: a systematic review. Cancer. 2021;13:5816. 10.3390/cancers13225816 PMC8616395 34830968

[4] Mehlis K , Bierwirth E , Laryionava K , et al. High prevalence of moral distress reported by oncologists and oncology nurses in end‐of‐life decision making. Psychooncology. 2018;27:2733‐2739. 30156350 10.1002/pon.4868

[5] Andrews C , Childers TC , Wiseman KD , et al. Facilitators and barriers to reducing chemotherapy for early‐stage breast cancer: a qualitative analysis of interviews with patients and patient advocates. BMC Cancer. 2022;22:1‐11. 35120494 10.1186/s12885-022-09189-w PMC8815019

[6] Rocque GB , Andrews C , Lawhon VM , et al. Oncologist‐reported barriers and facilitators to enrolling patients in optimization trials that test less intense cancer treatment. JCO Oncol Pract. 2023;19:e263‐e273. 36473142 10.1200/OP.22.00472

[7] Norton WE , Chambers DA , Kramer BS . Conceptualizing de‐implementation in cancer care delivery. J Clin Oncol. 2019;37:93‐96. 30407894 10.1200/JCO.18.00589

[8] Borgatti SP . Cultural domain analysis. J Quant Anthropol. 1994;4:261‐278.

[9] Borgatti SP . Elicitation Techniques for Cultural Domain Analysis. The Ethnographer's Toolkit, Vol. 3. Altimira Press; 1999:115‐151.

[10] Romney AK . Cultural consensus as a statistical model. Curr Anthropol. 1999;40:S93‐S115.

[11] Smith JJ , Borgatti SP . Salience counts‐and so does accuracy: correcting and updating a measure for free‐list‐item salience. J Linguist. 1997;7:208‐209.

[12] Wencelius J , Garine E , Raimond C . FLARES (free list analysis under R environment using shiny). wwwanthrocogscom/shiny/flares/ 2017.

[13] Weller SC , Vickers B , Bernard HR , et al. Open‐ended interview questions and saturation. PloS One. 2018;13:e0198606. 29924873 10.1371/journal.pone.0198606 PMC6010234

[14] Dressler WW , Balieiro MC , Dos Santos JE . Finding culture change in the second factor stability and change in cultural consensus and residual agreement. Field Methods. 2015;27:22‐38.

[15] Bennardo G , de Munck VC . Cultural Model Theory in Cognitive Anthropology: Recent Developments and Applications. Springer; 2020.

[16] de Munck VC , Bennardo G . Disciplining culture: a sociocognitive approach. Curr Anthropol. 2019;60:174‐193.

[17] Kronenfeld DB , Bennardo G , de Munck VC , et al. A Companion to Cognitive Anthropology. John Wiley & Sons; 2015.

[18] Bennardo G , Kronenfeld DB . Types of collective representations: cognition, mental architecture, and cultural knowledge. In: Kronenfeld DB , Bennardo G , de Munck V , et al., eds. A Companion to Cognitive Anthropology. John Wiley & Sons; 2015:82.

[19] Bennardo G , de Munck VC . Cultural Models: Genesis, Methods, and Experiences. Oxford University Press; 2014.

[20] Bennardo G , de Munck V , Kroger K , et al. A methodological trajectory to investigate cultural models: blending two approaches. 2014.

[21] Henderson NL , Monocello LT , Else RJ , Dressler WW . Modeling culture: a framework. Ethos. 2022;50:111‐130.

[22] Dressler WW , Balieiro MC , dos Santos JE . Distance from a cultural prototype and psychological distress in urban Brazil: a model. J Cogn Cult. 2023;23:218‐240.

[23] Wilt TJ , Brawer MK , Barry MJ , et al. The prostate cancer intervention versus observation trial: VA/NCI/AHRQ cooperative studies program# 407 (PIVOT): design and baseline results of a randomized controlled trial comparing radical prostatectomy to watchful waiting for men with clinically localized prostate cancer. Contemp Clin Trials. 2009;30:81‐87. 18783735 10.1016/j.cct.2008.08.002

[24] Vasileiou K , Barnett J , Thorpe S , Young T . Characterising and justifying sample size sufficiency in interview‐based studies: systematic analysis of qualitative health research over a 15‐year period. BMC Med Res Methodol. 2018;18:1‐18. 30463515 10.1186/s12874-018-0594-7 PMC6249736

